# ANN-Based Integrated Risk Ranking Approach: A Case Study of Contaminants of Emerging Concern of Fish and Seafood in Europe

**DOI:** 10.3390/ijerph18041598

**Published:** 2021-02-08

**Authors:** Vikas Kumar, Saurav Kumar

**Affiliations:** 1 Environmental Engineering Laboratory, Departament d’Enginyeria Química, Universitat Rovira i Virgili, 43007 Tarragona, Spain; sauravbioinfo3376@gmail.com; 2 IISPV, Hospital Universitari Sant Joan de Reus, Universitat Rovira i Virgili, 43201 Reus, Spain; 3 Department of Biochemistry, Deshbandhu College, University of Delhi, Delhi 110019, India

**Keywords:** self-organizing maps, hazard index, risk ranking, contaminants of emerging concern, seafood

## Abstract

Seafood, one of the most important food commodities consumed worldwide, is considered a high-quality, healthy, and safe food option. However, marine ecosystems are the ultimate destination for a large group of chemicals, including contaminants of emerging concern, and seafood consumption is a major pathway of human exposure. With growing awareness of food safety and food quality, and increased demand for information on the risk of contaminants of emerging concern, there is a need to assess food safety issues related to harmful contaminants in seafood and ensure the safety of marine food resources. In this study, the risks of emerging compounds (endocrine disruptors, brominated flame retardants, pharmaceuticals and personal care products, and toxic elements) in fish and seafood were analyzed according to their PBT (persistence, bioaccumulation, toxicity) properties as well as in terms of their concentration levels in seafood. A hazard index (HI) was estimated for each compound by applying an artificial neural network (ANN) approach known as Self-Organizing-Maps. Subsequently, an integrated risk rank (IRI) was developed considering the values of HI and the concentrations of emerging compounds in seafood species gathered from the scientific literature. Current results identified HHCB, MeHg, NP, AHTN and PBDE209 as the top five highest ranked compounds present in seafood, according to the 50th percentile (mean) of the IRI. However, this ranking slightly changed when taking into account the 99th percentile of the IRI, showing toxic elements, methylmercury and inorganic arsenic, as having the highest risk. The outcome of this study identified the priority contaminants and should help in regulatory decision-making and scientific panels to design screening programs as well as to take the appropriate safety measures.

## 1. Introduction

Seafood is one of the most important food commodities consumed worldwide, being recognized as a high-quality, healthy and safe food item. However, seafood consumption is also a relevant pathway of human exposure to environmental pollutants. The issue of seafood safety is even more important in view of the growth of the international fish trade, which has undergone a tremendous expansion in the last three decades, increasing from USD 8 billion in 1976, to a record export value of USD 102.5 billion in 2010 [1]. Consumption of seafood has also seen a continuous uptrend, with an average world consumption of 11.5 kg/capita/year in 1970 compared to 19.2 kg/capita/year in 2012 [2]. Therefore, safety of seafood is central to any society, and it has a wide range of economic, social and, in many cases, environmental consequences.

Marine ecosystems are the ultimate destination for a large group of chemicals, receiving these pollutants through rivers, direct discharges and atmospheric deposition. Fish and shellfish have been identified as the food group showing the highest concentrations of a number of toxic elements [3,4]. Some contaminants can bioaccumulate in marine organisms and biomagnify along the marine food web, likely being transferred to the human food chain, with subsequent potential problems for seafood safety [5,6]. Specifically, for seafood, maximum levels for a range of contaminants are outlined in the legislation, and seafood is regularly controlled by monitoring programs for a selection of environmental contaminants. This gives rise to concern from an environmental and public health point of view. So far, the focus mainly lays on well-known chemical pollutants, such as polycyclic aromatic hydrocarbons (PAHs), polychlorinated biphenyls (PCBs), certain marine toxins and some toxic elements [7,8,9,10,11]. However, there is no regulation in place for recently detected substances for which no maximum levels have been established in EU legislation and for which a potential risk cannot be excluded. Although it is not fully implemented yet, a new EC directive on priority substances in the field of water policy revised crucial rules on determining the chemical quality of surface water [12]. Furthermore, contaminants of emerging concern could be previously identified, for which maximum levels have been laid down but need revision due to new hazard information (re-emerging contaminants) [6]. Therefore, there is an increasing need for knowledge about the presence and potential effects of the so-called “contaminants of emerging concern” in seafood [6,13,14]. Special attention has been paid to pollutants belonging to four important groups of contaminants: toxic elements, endocrine disrupting compounds (EDCs), pharmaceuticals and personal care products (PPCPs), and brominated flame retardants (BRFs).

Toxic elements are widespread in the environment from either natural or anthropogenic sources [15]. Some of these elements can occur in food because of their presence in the environment or due to contamination during food production and storage. Some elements are essential to maintain a good health in humans but exposure to others can lead to severe adverse health effects [16]. Elements may change their chemical form in the environment, but they cannot be degraded over time. This means that they are environmentally persistent and may bioaccumulate [17,18]. The maximum levels of lead, cadmium and total mercury in seafood are regulated by the European Commission regulation 1881/2006 [7,8,9,19]. For other toxic elements or specific chemical forms, no maximum levels have been laid down in the European legislation, partly due to a lack of information about their presence in seafood. From a toxicological point of view, the chemical form (i.e., the elemental speciation) in which the metal is ingested plays a significant role [20,21]. Knowledge about the chemical form(s) of certain elements (e.g., inorganic arsenic: InAs and methyl mercury: MeHg) present in seafood is therefore required in order to improve the assessment of seafood safety beyond simply knowing the total elemental amount.

There is also a growing interest in EDCs due to their ability to interfere with the endocrine system of different organisms, causing important alterations in development. Because of the lipophilic and persistent nature of most EDCs and their metabolites, many of them can bioaccumulate and biomagnify in different environmental compartments, including in marine biota [22].

PPCPs are another diverse group of potential pollutants. They can enter aquatic ecosystems from municipal wastewater treatment plant discharges, runoff from agricultural areas that utilize veterinary therapeutics and releases from aquaculture sites [23,24]. As a result, they have been increasingly detected in the environment during recent years [25,26,27,28,29]. Another source of accumulation of PPCPs in fish and seafood is the prophylactic or therapeutic use of pharmaceuticals in aquaculture. Residues of these drugs can remain in tissues creating a potential exposure for consumers [24]. The presence of pharmaceuticals in seafood may potentially act as a risk for consumers, either through direct effect of allergy and toxicity or indirectly through potential microbial resistance [24].

Flame retardants comprise a large group of chemical substances that are widely used in many industrial and household products [30]. Currently, because of their high-performance efficiency and low cost, the largest market group of flame retardants is the brominated flame retardants (BFRs) group [31]. BFR-treated products, whether in use or waste, release BFRs into the environment. Unfortunately, these contaminants may then pass into the food chain causing toxic effects to human health [32,33,34,35,36,37,38,39].

Humans may potentially be exposed to emerging environmental contaminants by eating contaminated fish and seafood. However, monitoring the large group of contaminants of emerging concern is very extensive, so it is impossible to monitor all compounds. Considering the large list and a cost-effective use of resources, priorities for screening emerging contaminants in seafood should be set. Therefore, tools to combine and simplify large data collections are mandatory for risk managers and decision-makers. In recent years, many frameworks have been proposed to prioritize contaminants using a range of approaches classified as qualitative, semi-quantitative to quantitative methods [40,41]. Semi-quantitative risk assessment provides an intermediary level between the contextual evaluation of qualitative risk assessment and the numerical evaluation of quantitative risk assessment, by evaluating risks with a score [42,43]. A quantitative approach offers a more consistent and rigorous approach to assess and compare risks and risk management strategies, and avoids some of the greater ambiguities that a qualitative risk assessment may produce. However, qualitative approaches do not require the same mathematical skills and amount of data as quantitative risk assessments, which means they can be applied to risks and strategies where precise data are missing [42]. Recently, van der Fels-Klerx et al. [43] performed an extensive systematic literature review identifying and characterizing the available methodologies for risk ranking in the fields of feed and food safety. The following methods of risk ranking for chemical hazards were identified: risk assessment, risk ratio, scoring methods, risk matrices, multi criteria decision analysis (MCDA), flow charts/decision trees. Some of these methods are also classified as new approach methodologies (NAM) which have been recommended as complimentary tools for the integrated approach to testing and assessment (IATA) strategy [44,45].

Among these approaches, the relative scoring methods are the most widely reported approaches which allow ranking the list of chemical compounds by aggregating a selection of parameters. Relative scores indicate where a particular chemical stand within a specified normative sample of chemicals. For example, physicochemical parameters such as persistence, bioaccumulation and toxicity (PBT) are often used to build the hazard index (HI), a coefficient widely implemented to prioritize chemicals [46,47]. The applications of artificial neural network (ANN) or machine learning (ML) algorithms in chemical health and safety study can date back to the mid-1990s [48]. Most of these applications were in toxicity classification and prediction studies, however, lately these algorithms have also been used in hazardous property prediction and consequence analysis [44,49]. Ranking has traditionally been developed using various data aggregation methods such as partial order ranking [50], utility function or simple additive ranking [51], fuzzy-based risk [52,53], Bayesian network classification [54,55], and clustering based ANN methods, such as Self-Organizing-Maps (SOM) [20,47]. In the latter case, HI is determined by intrinsic parameters of the chemicals (PBT) and risk can be described as a function of hazard (toxicity) and exposure (dose). Due to the ability to group data according to similar characteristics, the SOM algorithm was previously used to create PBT-based rankings of chemical pollutants [20,47,56]. Recently, SOM was also applied to elaborate an ecological hazard index of a series of pollutants found in Ebro River waters (Spain) [52,53]. Integration of PBT parameters with exposure levels in target food groups can be an interesting approach to obtain realistic information for food safety policies.

The objective of the present study was to prioritize a selection of contaminants of emerging concern by means of an artificial neural network (ANN) based approach integrating PBT properties and the concentration levels of these pollutants in seafood species. Firstly, HI was generated and applied to each individual compound by using SOM. Secondly, an integrated risk ranking was developed by combining the HI and concentration level of each compound in seafood, considering the linearity between concentration levels in the food source and the possible dose. Finally, a prioritized list of emerging contaminants was performed by ranking the chemicals according to the integrated risk score.

## 2. Materials and Methods

### 2.1. Raw Data Sets

#### 2.1.1. List of Chemicals

A list of 62 emerging chemicals was elaborated according to the availability of concentration data on seafood species in the ECsafeSEAFOOD database [6] (Table 1). Chemicals from four important groups of contaminants were incorporated in this study: toxic elements (*n* = 2), EDCs (*n* = 19), PPCPs (*n* = 31), and BFRs (*n* = 10).

#### 2.1.2. PBT Parameters

The values of three parameters (persistence, bioaccumulation and toxicity) were assembled from the quantitative structure–activity relationship (QSAR) modelling software Estimation Program Interface (EPI Suite^TM^, [57]). EPI Suite is a Windows based software developed by the Office of Pollution Prevention Toxics and Syracuse Research Corporation (SRC), U.S. Environmental Protection Agency (EPA) [57]. This screening-level tool is used to estimate the physical and chemical properties, environmental fate and aquatic toxicology of chemicals, integrating data of more than 41,000 compounds from the PHYSPROP© database (Syracuse Research Corporation, Syracuse, NY, USA). It is a very powerful tool used to obtain estimated values when experimental information is not available.

(a) Persistence: environmental half-lives of each chemical were estimated using the Biowin^TM^ tool [57], capable of predicting the primary aerobic and anaerobic biodegradability of organic chemicals using 7 different models, the results of which were reconverted to a semi-quantitative rate of times, with the following units: 5 h, 4 days, 3 weeks, 2 months, and 1 year [58].
(b) Bioaccumulation: bioconcentration factor logarithm (log BCF) was obtained from BCFWin^TM^ [57] through the octanol–water constant (K_ow_).
(c) Toxicity: toxicity was estimated through the Ecological Structure Activity Relationships (EcoSAR^TM^, [57]) tool which estimates acute and chronic toxicity to aquatic organisms of different trophic levels: fish, aquatic invertebrates and green algae (Sanderson et al., 2003). The toxicity data used to build the SARs were collected from publicly available experimental studies and confidential submissions provided to the U.S. EPA New Chemicals Program.

#### 2.1.3. Contamination Levels

The information currently available about emerging environmental contaminants is rather dispersed. A database was developed [6], to compile all the information from the scientific literature concerning emerging contaminant levels in seafood. Based on the information available in this database, the mean and range of the concentrations of each one of the pollutants in seafood, was estimated. Only studies reporting concentration levels expressed in wet weight were considered, as conversion factors from dry or lipid to wet weight, were not available for all species. Thus, for each one of the 62 contaminants, distribution data of concentration levels (in wet weight) in marine fish, mollusks and crustaceans were gathered and a minimum, mean and maximum concentration was reported (Table 2). Because of unavailability of PPCP data on marine biota, studies on freshwater biota were used. As data on contamination levels in European seafood are scarce, non-European studies were also included in this study. Because this study is assessing the risk of consumption of seafood, only edible fractions were considered. For fish and crustaceans only levels in meat were considered. Levels in liver/gonads/blood of fish and in hepatopancreas/gonads of crustaceans were not considered. For mollusks, levels in the whole body were used.

### 2.2. Hazard Index

The compilation and organization of large amounts of data can be computed by data mining tools, such as ANNs [59]. Among the different kinds of ANNs, Kohonen’s Self-Organising-Map (SOM) is one of the most commonly applied methods [60]. SOM uses an unsupervised learning algorithm that reduces the dimensionality of large input data and utilizes a neighborhood function to preserve the topological properties of the input space [61]. The results are generally visualized in two-dimension maps, allowing for clustering of the input information by grouping similar data characteristics. The final result, is on the one hand, a low dimension map (or Kohonen’s map) showing the discretized representation of the multidimensional input space, and on the other hand, a set of component planes showing the clusters created by the algorithm in the Kohonen’s grid. The ability of SOM to group data and cluster the analyzed parameters has been extensively applied in environmental toxicology, but little is known about its applicability in food toxicology [58,62,63,64,65,66]. The interpretation of the SOM clusters begins with the map visualization. Each of the SOM nodes (neuron or hexagonal grid) has a specific weight, allowing one to cluster the original information, akin to multidimensional scaling. The weights associated to each node or neuron in a two-dimensional lattice are adjusted to cluster the original information. The map can also be divided into so many c-planes (component planes) as data variables, representing the variable contribution to each node in the map [20].

The integration of PBT parameters was performed with inbuilt functions of SOM toolbox for Matlab^TM^. The HI for each chemical was built by integration of PBT parameters through the distance measure (such as Euclidean distance), which is the average distance between the node’s weight vector and that of its closest neighbors used in Kohonen’s algorithm. A linear initialization was applied for SOM clustering. The competitive learning phase consisted of 10,000 steps, while the tuning phase added another 10,000 steps. After iterative trainings, SOM is eventually formed in the format that inputs with similar features are mapped to the same map unit or nearby neighboring units, creating a smooth transition of related individuals over the entire map. HI was considered as the sum of the PBT values for each compound after SOM training. As low levels of persistence and toxicity result in a higher hazard, inverse values obtained from the BiowinTM and EcoSARTM tools, respectively, were considered in the HI building [58]. The three full datasets were normalized to obtain a variance equal to one for each parameter. Default ranges of PBT parameters was re-scaled to 0–10 and hazard indexes were normalized using Equation (1) and re-scaled from 0 to 10.

(1) $$C_{norm}=\frac{C_{i}-C_{min}}{C_{max}-C_{min}}$$

where *C_norm_* is the normalized value, *C_i_* is the parameter value of species *i*, *C_min_* is the lowest and *C_max_* is the maximum concentration value.

### 2.3. Risk Ranking

In order to apply weights to the effects of contamination levels, PBT parameters were integrated with the distribution data of concentration levels of pollutants in seafood as follows (Equation (2)):

(2) $$RI_{t}\left(Mean\pm Std\right)=(HI_{t})\times \left(P\langle C_{t,s}\left(\mu_{t},\sigma_{t}\right)\rangle \right)$$

where *RI*_t_ is the risk index for the contaminant *t*, *HI*_t_ is the hazard index for the contaminant *t* and *C*_t,s_ is the *s*th sample of concentration level of the contaminant *t* in seafood from the sample concentration generated with mean $\mu_{t}$ standard deviation $\sigma_{t}$. Uncertainties of the concentration have been included in the risk ranking calculation by simulating the concentration distribution. If a mean concentration (and standard deviation) of the contaminant was available in seafood, a normal data distribution was assumed. If only the min–max range was available, data distribution was assumed to be uniform. Mean and standard deviation of the risk index was calculated and reported in Table 3.

## 3. Results and Discussion

**Figure 1:**
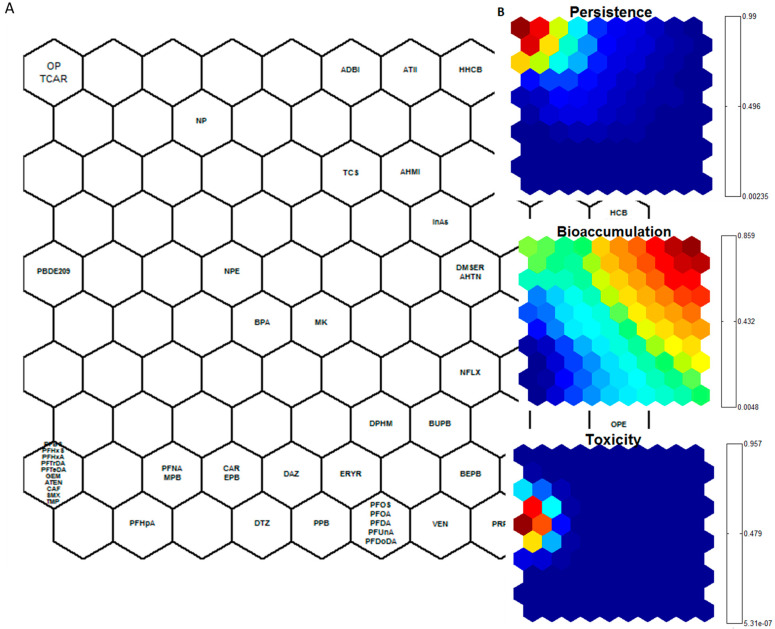
Self-Organizing-Maps of the hazard index (HI): (**A**) Labeled cluster of Kohonen’s map; (**B**) c-planes of PBT. Grouping based on PBT parameters (persistence, bioaccumulation and toxicity) values of the pollutants under study. For definitions of compound abbreviations refer to Table 1. Color intensity shows numeric strength of index, the brighter the color, the higher the value. The label display shows positions of each unit on the map.

The application of the SOM clustering algorithm to PBT data of all the compounds listed in Table 4 has resulted in grouping of chemicals based on their PBT properties. The clustering map structure was based on a two-dimensional grid of 100 (10 × 10) cells. The data training phase consists of a twostep primary training and a tuning phase. The labeled Cluster of Kohonen’s map (Figure 1A) visualizes distances between neighboring map units, and thus shows the cluster structures of the map obtained from iterative process of unsupervised learning. The C-planes show the distance measure obtained from normalized values of persistence, bioaccumulation and toxicity obtained from an iterative SOM procedure (Figure 1B). The color-coding index stands for the normalized integrated values of the respective chemical properties. The color index of each display was established on the basis of all the values of a single component plane. All these presentations are linked by position: in each display, the hexagon in a certain position corresponds to the same map unit. Color intensity shows the numeric strength of the index, while the label display shows positions of each unit on the map.

The SOM based HI for each emerging contaminant is summarized in Table 5 and ranked according to its absolute score. Considering the HI values, the highest scores were attributed to BDE209 (4.473), octylphenol (4.367), triclocarban (4.367), MeHg (2.625), tetrabromobisphenol A (2.625), BDE47 (2.504), perfluorooctanesulfonamide (2.493), and hexabromocyclododecane (2.493). While BDE209 was identified as the most toxic compound, high HI values of the remaining top compounds were due to their high bioaccumulation (MeHg, TBBPA, PFOSA, BDE47, HBCD) or persistence (OP, TCAR).

Both toxic elements, MeHg and InAs, were found in the upper side of the ranking. PPCPs and EDCs are evenly distributed throughout the ranking, while BFRs reached the highest scores as a group. The BCF cluster map clearly provided three clusters, while those relative to persistence and toxicity entailed two main clusters (Figure 1B). As the Kohonen’s based HI is a mutual scoring method where scores are computed using a set of properties data, the comparisons with other studies become complicated. Nonetheless, Fabrega et al. [58] implemented a similar methodology on PPCPs, EDCs, pesticides, perfluorinated compounds (PFCs), illicit drugs and UV filters. In that study, the most hazardous pollutants were identified to be six PFCs (PFHxDA, PFODA, PFTeDA, PFTrDA, PFDoA, and PFUdA).

**Figure 2:**
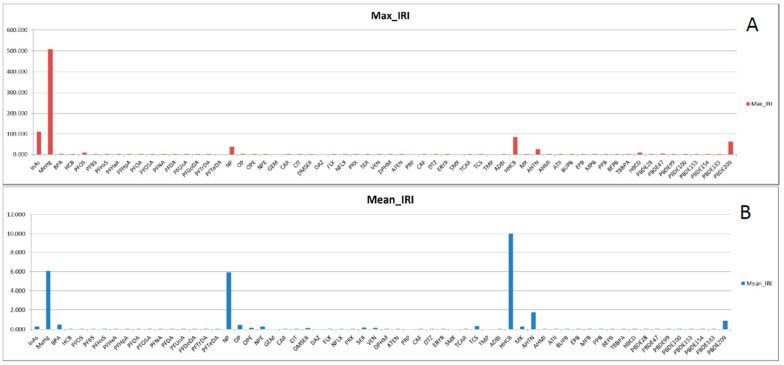
Integrated risk index of (**A**) max (99 percentile) and (**B**) mean (50 percentile) of integrated risk index score.

**Figure 3:**
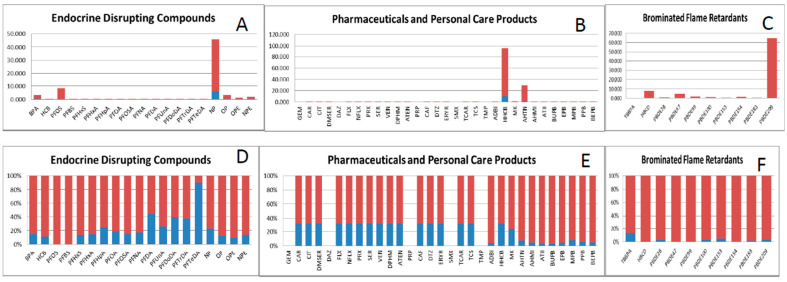
Integrated risk index of different group of compound showing (**A**–**C**) the ratio of mean (50 percentile) and max (99 percentile) IRI score and (**D**–**F**) the percentage of mean (50 percentile) and max (99 percentile) score.

Since the PBT based HI values do not reflect the current situation in terms of consumer safety, we implemented a second step by integrating the HI with the concentration of these emerging compounds in commercial seafood. This integration was performed by multiplying the HI score with the concentration level of the compounds in seafood (as explained in Section 2.3), weighting the contamination vector in the final score. Since the concentration data account for the distributional variability of different samples reported in the literature, the risk index is calculated as mean and standard deviation by propagating the uncertainty of concentration in the integrated risk index (IRI) calculation. The resulting integrated index provides a new ranking of chemicals considering the current contamination of seafood reported as mean ± Std (Table 3). In the overall ranking based on maximum value (99 percentile) of the IRI score, metals (MeHg and InAs) occupy the highest rank followed by HHCB, BDE209, AHTN and NP belonging to different groups of compounds (Figure 2A). Nonylphenol (NP), perfluorinated octane sulfonate (PFOS) and bisphenol A (BPA) (Figure 3A) were the endocrine disrupting compounds with highest risk, whereas most PFCs showed the lowest risk index due to their low concentration values (Table 2). Among the PPCPs, galaxolide (HHCB) and tonalide (AHTN) (Figure 3B) were estimated to be the riskiest contaminants. However, since the PPCPs risk index was calculated using concentration values in freshwater biota, an overestimation may have occurred. In the BFRs group, BDE209, HBCD and BDE47 (Figure 3C) were ranked as the flame retardants with the highest risk.

Uncertainty is inherent in the process even when using the most accurate data and the most sophisticated models. However, in this case, only uncertainty due to concentration variability was considered. “Uncertainty” is, in this case, the description of the imperfect knowledge of the true value of concentration level, or its real variability in samples or observations. The dataset for this study is very heterogeneous with different sample sizes and it is based on reported values in the literature [6]. The degree of uncertainty of the IRI was analyzed using linear propagation of uncertainty of concentration levels (Equation (2)). Using mean values of IRI (low degree of conservatism), the risk ranking of these compounds changed significantly, in comparison to the 99th percentile of the IRI (high degree of conservatism). In the overall ranking based on mean values (50th percentile) of the IRI score, HHCB occupied the highest position, followed by MeHg, NP, AHTN and BDE209 in the top five ranked compounds (Figure 2B). Detailed IRI scores with the mean and standard deviation of all compounds in this study are presented in Table 3. Comparing the ratio of mean and max values of IRI score (Figure 3D–F), it is evident that IRI score is skewed towards the higher end of the distribution, which is mainly due to small sample of concentration data biased toward extreme outliers (higher concentration value).

A limitation of the method used in this study is the use of theoretical values as HI parameters. Since this study focuses on contaminants of emerging concern, data on persistence, bioaccumulation and toxicity are unavailable in the scientific literature for most compounds and were thus estimated by applying the US EPI Suit^TM^ software [57]. The process of modeling PBT data may be associated with uncertainty, especially for the “toxicity” variable. In this study, only fish toxicity values were used to estimate the HI by means of the ECOSAR^TM^ tool [57]. This may lead to a significant bias, as the relationship with human toxicity is not taken into account. In this framework, further improvements of the hazard index should be focused on incorporating experimental PBT values whenever new data become available.

Another important limitation in this study is the exposure parameter. Since dietary exposure is determined by both concentration and consumption, the dietary consumption vector should be considered as an additional parameter in future studies. In this study, the complexity of the database does not allow for the provision of an individual input for each pollutant and fish species. However, the mean level of consumption for all the species (as mean European fish level intake) will be the same through the different pollutants and will not affect the risk index. Moreover, contamination levels considered in this study were averaged from all seafood species, sometimes including freshwater species, as the availability of levels of contaminants of emerging concern was limited. A specific ranking for each species should be performed in future studies once the levels of every contaminant become available for each seafood species.

## 4. Conclusions

Risk ranking frameworks for chemical hazards have been mainly developed to establish priority settings in order to reduce environmental problems related with pollutants, as well as to provide an objective tool for risk managers and decision makers for resources optimization. It also provides a user-friendly visualization and data analysis approach to be used as a risk communication and management strategy. The objectivity of risk ranking has been improved by applying a quantitative approach in the form of a SOM based methodology for risk ranking contaminants of emerging concern in food safety. By combining HI based on PBT parameters with contamination levels in seafood, the IRI was estimated for each environmental pollutant using SOM. The highest HI values were estimated for BDE209, octylphenol, triclocarban, MeHg, tetrabromobisphenol A, BDE47, perfluorooctanesulfonamide, and hexabromocyclododecane. Nonetheless, the integration of concentration levels with the HI modified this ranking, resulting in HHCB, MeHg, NP, AHTN and BDE209 emerging as the top five ranked compounds, according to the 50th percentile (mean) score of IRI. Furthermore, and considering the 99th percentile of IRI score, the risk ranking slightly changed, with toxic elements (MeHg and InAs) posing the highest risk, followed by HHCB, BDE209, AHTN and NP.

Uncertainty is introduced at every step of the health risk assessment. Unfortunately, in this particular case, the uncertainty associated with PBT values was not accounted due to the scarce information in the QSAR model for emerging contaminants. The availability of homogeneous high-quality data can determine the accuracy and uncertainties associated with the final results of this method. As information on PBT values and contamination levels in seafood is very heterogeneous and scarce for contaminants of emerging concern, theoretical values need to be used. Further improvements on the use of this method should be focused on incorporating homogeneous experimental values and model the uncertainty of PBT values. Besides these improvements, other aspects such as consumption levels, could be added to improve the risk ranking method, while other emerging pollutants should ideally also be incorporated.

## Figures and Tables

**Table 1:** List of emerging pollutants included in the risk ranking.

Group	CAS Number	Compound	Label
Toxic elements	7440-38-2 (arsenic)	Inorganic Arsenic	InAs
	22967-92-6	Methyl mercury	MeHg
Endocrine Disrupting Compounds (EDCs)	80-05-7	Bisphenol A	BPA
	118-74-1	Hexachlorobenzene	HCB
	1763-23-1	Perfluorinated octane sulfonate	PFOS
	375-73-5	Perfluorobutanesulfonic acid	PFBS
	355-46-4	Perfluorohexane sulfonic acid	PFHxS
	307-24-4	Perfluorohexanoic acid	PFHxA
	375-85-9	Perfluoroheptanoic acid	PFHpA
	335-67-1	Perfluorinated octanoic carboxylic acid	PFOA
	754-91-6	Perfluorooctanesulfonamide	PFOSA
	375-95-1	Perfluorononanoic acid	PFNA
	335-76-2	Perfluorodecanoic acid	PFDA
	2058-94-8	Perfluoroundecanoic acid	PFUnA
	307-55-1	Perfluorododecanoic acid	PFDoDA
	72629-94-8	Perfluorotridecanoic acid	PFTrDA
	376-06-7	Perfluorotetradecanoic acid	PFTeDA
	number of isomeric compounds	Nonylphenol	NP
	number of isomeric compounds	Octylphenol	OP
	number of isomeric compounds	OctylphenolMonoethoxylate	OPE
	number of isomeric compounds	NonylphenolMonoethoxylate	NPE
Pharmaceuticals and Personal Care Products (PPCPs)	25812-30-0	Gemfibrozil	GEM
	298-46-4	Carbamazepine	CAR
	59729-32-7	Citalopram	CIT
	87857-41-8	Desmethylsertraline	DMSER
	439-14-5	Diazepam	DAZ
	54910-89-3	Fluoxetine	FLX
	83891-03-6	Norfluoxetine	NFLX
	61869-08-7	Paroxetine	PRX
	79617-96-2	Sertraline	SER
	93413-69-5	Venlafaxine	VEN
	58-73-1	Diphenhydramine	DPHM
	29122-68-7	Atenolol	ATEN
	525-66-6	Propanolol	PRP
	58-08-2	Caffeine	CAF
	42399-41-7	Diltiazem	DTZ
	114-07-8	Erythromycine	ERYR
	723-46-6	Sulfamethoxazole	SMX
	101-20-2	Triclocarban	TCAR
	3380-34-5	Triclosan	TCS
	738-70-5	Trimethoprim	TMP
	13171-00-1	Celestolide	ADBI
	1222-05-5	Galaxolide	HHCB
	81-14-1	Musk ketone	MK
	21145-77-7	Tonalide	AHTN
	15323-35-0	Phantolide	AHMI
	68140-48-7	Traseolide	ATII
	94-26-8	Butylparaben	BUPB
	120-47-8	Ethylparaben	EPB
	99-76-3	Methylparaben	MPB
	94-13-3	Propylparaben	PPB
	94-18-8	Benzylparaben	BEPB
Brominated Flame Retardants (BFRs)	79-94-7	Tetrabromobisphenol A	TBBPA
	25637-99-4	Hexabromocyclododecane	HBCD
	41318-75-6	2,4,4Tribromodiphenyl ether	PBDE28
	5436-43-1	2,2′,4,4′-Tetra-	PBDE47
		bromodiphenyl ether	
	60348-60-9	2,2′,4,4′,5-Penta-	PBDE99
		bromodiphenyl ether	
	189084-64-8	2,2′,4,4′,6-Penta-	PBDE100
		bromodiphenyl ether	
	68631-49-2	2,2′,4,4′,5,5′-Hexa-	PBDE153
		bromodiphenyl ether	
	207122-15-4	2,2′,4,4′,5,6′-Hexa-	PBDE154
		bromodiphenyl ether	
	207122-16-5	1,2,3,5-tetrabromo-4-(2,4,5-tribromophenoxy)benzene	PBDE183
	1163-19-5	1,2,3,4,5-pentabromo-6-(2,3,4,5,6-pentabromophenoxy)benzene	PBDE209

**Table 2:** Concentration values of selected emerging contaminants in seafood species (units in ng/g wet weight).

Contaminant	Concentartion (ng/g ww)			Contaminant	Concentartion (ng/g ww)		
	Min	Mean	Max		Min	Mean	Max
InAs	2	14.10	5800	DPHM	0	1.25	2.5
MeHg	0	230.00	19,370	ATEN	0	0.15	0.3
BPA	0	39.40	233.3	PRP	0	-	0
HCB	0.006	0.21	1.68	CAF	0	2.25	4.5
PFOS	0	1.71	877	DTZ	0	0.14	0.27
PFBS	0	0.01	13.45	ERYR	0	0.05	0.1
PFHxS	0.003	0.09	0.52	SMX	0	-	0
PFHxA	0.004	0.07	0.39	TCAR	0	0.75	1.5
PFHpA	0.082	0.14	0.43	TCS	0	15.50	31
PFOA	0.078	0.76	3.25	TMP	0	-	0
PFOSA	0.0378	0.53	2.957	ADBI	0	0.78	18.3
PFNA	0	0.21	1.02	HHCB	0	416.00	3600
PFDA	0	0.24	0.3	MK	0	22.90	73
PFUnA	0.024	0.32	0.93	AHTN	0	96.20	1500
PFDoDA	0.001	0.16	0.24	AHMI	0	1.16	21.5
PFTrDA	0.15	0.28	0.46	ATII	0	0.57	15.4
PFTeDA	0	1.25	0.14	BUPB	0	0.01	0.269
NP	0	242.00	1639	EPB	0	0.11	2.27
OP	0	9.66	66.1	MPB	0	1.09	12.2
OPE	0	8.73	78	PPB	0	0.09	1.48
NPE	1	21.10	127.7	BEPB	0	0.13	2.47
GEM	0	-	0	TBBPA	0	0.30	1.9
CAR	0	2.65	5.3	HBCD	0	0.61	329
CIT	0	2.11	4.21	BDE28	0	1.39	38
DMSER	0	6.00	12	BDE47	0	0.51	197
DAZ	0	-	0	BDE99	0	0.59	91
FLX	0	3.30	6.6	BDE100	0	1.87	56
NFLX	0	2.50	5	BDE153	0	1.23	24
PRX	0	0.29	0.58	BDE154	0	1.03	94
SER	0	9.50	19	BDE183	0	0.55	25
VEN	0	15.00	30	BDE209	0	19.20	1433

**Table 3:** Ranking of emerging contaminants based on mean (50 percentile) integrated risk rank (IRI) score considering the persistence, bioaccumulation, toxicity (PBT) parameters and concentration levels in seafood.

Ranking Position	Contaminant	Integrated Risk Index (Mean ± Std)	Ranking Position	Contaminant	Integrated Risk Index (Mean ± Std)
1	HHCB	1.00 × 10^1^ ± 2.50 × 10^1^	32	BDE47	1.26 × 10^−2^ ± 1.42
2	MeHg	6.04 ± 1.47 × 10^2^	33	BDE99	1.26 × 10^−2^ ± 5.64 × 10^−1^
3	NP	5.92 ± 1.16 × 10^1^	34	BDE183	9.99 × 10^−3^ ± 1.31 × 10^−1^
4	AHTN	1.76 ± 7.92	35	TBBPA	7.87 × 10^−3^ ± 1.44 × 10^−2^
5	BDE209	8.59 × 10^−1^ ± 1.85 × 10^1^	36	PFOA	7.09 × 10^−3^ ± 8.59 × 10^−3^
6	BPA	4.76 × 10^−1^ ± 8.14 × 10^−1^	37	PRX	4.97 × 10^−3^ ± 2.87 × 10^−3^
7	OP	4.22 × 10^−1^ ± 8.33 × 10^−1^	38	HCB	4.42 × 10^−3^ ± 1.04 × 10^−2^
8	TCS	3.07 × 10^−1^ ± 1.77 × 10^−1^	39	MPB	3.89 × 10^−3^ ± 1.26 × 10^−2^
9	MK	2.82 × 10^−1^ ± 2.59 × 10^−1^	40	PFUnA	3.02 × 10^−3^ ± 2.45 × 10^−3^
10	NPE	2.76 × 10^−1^ ± 4.78 × 10^−1^	41	PFDA	2.28 × 10^−3^ ± 8.12 × 10^−4^
11	InAs	2.71 × 10^−1^ ± 3.22 × 10^1^	42	PFDoDA	1.49 × 10^−3^ ± 6.47 × 10^−4^
12	SER	1.88 × 10^−1^ ± 1.09 × 10^−1^	43	BEPB	1.44 × 10^−3^ ± 7.98 × 10^−3^
13	VEN	1.51 × 10^−1^ ± 8.69 × 10^−2^	44	DTZ	9.52 × 10^−4^ ± 5.50 × 10^−4^
14	OPE	1.40 × 10^−1^ ± 3.61 × 10^−1^	45	CAF	9.04 × 10^−4^ ± 5.22 × 10^−4^
15	DMSER	1.10 × 10^−1^ ± 6.33 × 10^−2^	46	PPB	8.02 × 10^−4^ ± 3.68 × 10^−3^
16	FLX	4.54 × 10^−2^ ± 2.62 × 10^−2^	47	PFNA	7.50 × 10^−4^ ± 1.05 × 10^−3^
17	BDE100	4.01 × 10^−2^ ± 3.47 × 10^−1^	48	EPB	6.52 × 10^−4^ ± 3.88 × 10^−3^
18	NFLX	3.64 × 10^−2^ ± 2.10 × 10^−2^	49	PFTeDA	5.02 × 10^−4^ ± 1.62 × 10^−5^
19	TCAR	3.28 × 10^−2^ ± 1.89 × 10^−2^	50	ERYR	4.57 × 10^−4^ ± 2.64 × 10^−4^
20	BDE28	3.18 × 10^−2^ ± 2.51 × 10^−1^	51	PFHpA	3.19 × 10^−4^ ± 2.24 × 10^−4^
21	CIT	2.59 × 10^−2^ ± 1.50 × 10^−2^	52	PFTrDA	1.12 × 10^−4^ ± 3.59 × 10^−5^
22	AHMI	2.34 × 10^−2^ ± 1.25 × 10^−1^	53	BUPB	1.06 × 10^−4^ ± 9.16 × 10^−4^
23	BDE153	2.23 × 10^−2^ ± 1.26 × 10^−1^	54	ATEN	6.02 × 10^−5^ ± 3.48 × 10^−5^
24	BDE154	1.87 × 10^−2^ ± 4.93 × 10^−1^	55	PFHxS	3.47 × 10^−5^ ± 5.99 × 10^−5^
25	ADBI	1.69 × 10^−2^ ± 1.14 × 10^−1^	56	PFHxA	2.62 × 10^−5^ ± 4.47 × 10^−5^
26	PFOS	1.60 × 10^−2^ ± 2.37	57	PFBS	3.41 × 10^−6^ ± 1.56 × 10^−3^
27	CAR	1.57 × 10^−2^ ± 9.06 × 10^−3^	58	GEM	0.00 ± 0.00
28	HBCD	1.52 × 10^−2^ ± 2.37	58	DAZ	0.00 ± 0.00
29	PFOSA	1.31 × 10^−2^ ± 2.10 × 10^−2^	58	PRP	0.00 ± 0.00
30	DPHM	1.29 × 10^−2^ ± 7.44 × 10^−3^	58	SMX	0.00 ± 0.00
31	ATII	1.28 × 10^−2^ ± 1.00 × 10^−1^	58	TMP	0.00 ± 0.00

**Table 4:** Persistence, bioaccumulation and toxicity parameters for the 62 emerging contaminants.

	Persistence	Bioaccumulation	Toxicity
	Log Days	Log L/Kg	mg/L
InAs	2.87	0.50	3.00 × 10^−3^
MeHg	1.82	2.00	1.00 × 10^−4^
BPA	0.58	1.86	1.28
HCB	2.44	3.45	8.00 × 10^−4^
PFOS	1.95	1.75	2.37 × 10^1^
PFBS	2.86	0.50	3.60 × 10^3^
PFHxS	2.41	0.50	3.01 × 10^2^
PFHxA	2.89	0.50	1.22 × 10^2^
PFHpA	2.67	0.75	3.54 × 10^1^
PFOA	2.44	1.75	1.01 × 10^1^
PFOSA	1.78	3.84	1.58 × 10^−1^
PFNA	2.22	1.00	2.84
PFDA	2.08	1.75	7.88 × 10^−1^
PFUnA	2.41	1.75	2.17 × 10^−1^
PFDoDA	2.74	1.75	5.90 × 10^−2^
PFTrDA	1.31	0.50	1.60 × 10^−2^
PFTeDA	1.09	0.50	4.00 × 10^−3^
NP	0.12	2.09	3.60 × 10^−2^
OP	0.05	1.92	7.90 × 10^−2^
OPE	3.31	2.69	1.15
NPE	0.34	1.72	1.33 × 10^−1^
GEM	3.66	0.50	6.73 × 10
CAR	3.51	1.28	1.16 × 10^2^
CIT	2.88	2.14	7.26 × 10
DMSER	1.31	2.85	7.87 × 10^−1^
DAZ	3.48	1.53	5.50 × 10^1^
FLX	3.25	2.34	1.06
NFLX	3.27	2.43	2.66
PRX	3.59	2.80	9.29 × 10^−1^
SER	1.66	3.16	2.81 × 10^−1^
VEN	3.01	1.83	1.61 × 10^1^
DPHM	1.32	1.83	2.13 × 10^1^
ATEN	3.85	0.50	1.44 × 10^4^
PRP	3.72	1.96	6.19 × 10^1^
CAF	3.61	0.50	7.22 × 10^3^
DTZ	3.47	1.45	6.58 × 10^1^
ERYR	2.59	1.69	2.24 × 10^2^
SMX	3.31	0.50	4.78 × 10^3^
TCAR	0.05	2.90	6.42 × 10^−1^
TCS	0.52	2.81	9.65 × 10^−1^
TMP	3.37	0.50	3.30 × 10^3^
ADBI	0.44	2.99	6.00 × 10^−2^
HHCB	0.55	3.56	3.20 × 10^−2^
MK	0.76	1.92	2.02
AHTN	1.05	2.84	2.70 × 10^−2^
AHMI	0.58	2.94	6.90 × 10^−2^
ATII	0.51	3.25	2.90 × 10^−2^
BUPB	1.18	2.02	7.63
EPB	1.53	1.30	4.98 × 10^1^
MPB	1.68	0.96	1.26 × 10^2^
PPB	3.86	1.67	1.96 × 10^1^
BEPB	3.79	2.02	5.52
TBBPA	2.11	4.03	2.30 × 10^−2^
HBCD	1.49	3.76	4.00 × 10^−3^
BDE28	1.36	3.55	1.10 × 10^−1^
BDE47	2.61	3.83	2.10 × 10^−2^
BDE99	2.34	3.39	4.00 × 10^−3^
BDE100	2.34	3.39	4.00 × 10^−3^
BDE153	2.08	2.95	6.93 × 10^−4^
BDE154	2.08	2.95	6.93 × 10^−4^
BDE183	2.16	2.93	1.24 × 10^−4^
BDE209	1.01	1.21	6.56 × 10^−7^

**Table 5:** Hazard Index (HI) developed for the 62 compounds by means of the Kohonen’s algorithm.

Ranking Position	Contaminant	HI	Ranking Position	Contaminant	HI
1	BDE209	4.473	23	MK	1.230
2	OP	4.367	24	BPA	1.208
2	TCAR	4.367	25	BUPB	1.180
3	MeHg	2.625	26	BEPB	1.119
3	TBBPA	2.625	27	PRP	1.092
4	BDE47	2.504	28	DPHM	1.031
5	PFOSA	2.493	29	VEN	1.004
5	HBCD	2.493	30	PFOS	0.938
6	NP	2.448	30	PFOA	0.938
7	HHCB	2.403	30	PFDA	0.938
8	BDE28	2.290	30	PFUnA	0.938
9	ATII	2.251	30	PFDoDA	0.938
10	ADBI	2.166	31	ERYR	0.914
11	HCB	2.146	32	PPB	0.862
11	BDE99	2.146	33	DAZ	0.780
11	BDE100	2.146	34	DTZ	0.705
12	AHMI	2.019	35	CAR	0.592
13	TCS	1.981	35	EPB	0.592
14	SER	1.980	36	PFNA	0.357
15	InAs	1.922	36	MPB	0.357
16	DMSER	1.829	37	PFHpA	0.223
16	AHTN	1.829	38	PFBS	0.040
17	BDE153	1.817	38	PFHxS	0.040
17	BDE154	1.817	38	PFHxA	0.040
17	BDE183	1.817	38	PFTrDA	0.040
18	PRX	1.716	38	PFTeDA	0.040
19	OPE	1.603	38	GEM	0.040
20	NFLX	1.457	38	ATEN	0.040
21	FLX	1.377	38	CAF	0.040
22	NPE	1.306	38	SMX	0.040
23	CIT	1.231	38	TMP	0.040

## Data Availability

The data presented in this study are available on request from the corresponding author.
